# Nonionic surfactant attenuates acute lung injury by restoring epithelial integrity and alveolar fluid clearance

**DOI:** 10.7150/ijms.51905

**Published:** 2021-01-21

**Authors:** Po-Chun Hsieh, Chan-Yen Kuo, Chin-Pyng Wu, Chung-Tai Yue, Chung-Kan Peng, Kun-Lun Huang, Chou-Chin Lan

**Affiliations:** 1 Department of Chinese Medicine, Taipei Tzu Chi Hospital, Buddhist Tzu Chi Medical Foundation, New Taipei City, Taiwan.; 2 Graduate Institute of Medical Sciences, National Defense Medical Center, Taipei, Taiwan.; 3 Department of Research, Taipei Tzu Chi Hospital, Buddhist Tzu Chi Medical Foundation, New Taipei City, Taiwan.; 4 Department of Critical Care Medicine, Landseed International Hospital, Tao-Yuan City, Taiwan.; 5 Department of Anatomic Pathology, Taipei Tzu Chi Hospital, Buddhist Tzu Chi Medical Foundation, New Taipei City, Taiwan; Department of Pathology, Buddhist Tzu Chi University, Hualien, Taiwan.; 6 Division of Pulmonary Medicine, Tri-Service General Hospital, Taipei, Taiwan; Institute of Undersea and Hyperbaric Medicine, National Defense Medical Center, Taipei, Taiwan.; 7 Division of Pulmonary Medicine, Taipei Tzu Chi Hospital, Buddhist Tzu Chi Medical Foundation; School of Medicine, Tzu-Chi University, Hualien, Taiwan.

**Keywords:** nonionic surfactant, air emboli, epithelial integrity, lung injury

## Abstract

**Introduction:** Acute lung injury (ALI) has a great impact and a high mortality rate in intensive care units (ICUs). Excessive air may enter the lungs, causing pulmonary air embolism (AE)-induced ALI. Some invasive iatrogenic procedures cause pulmonary AE-induced ALI, with the presentation of severe inflammatory reactions, hypoxia, and pulmonary hypertension. Pulmonary surfactants are vital in the lungs to reduce the surface tension and inflammation. Nonionic surfactants (NIS) are a kind of surfactants without electric charge on their hydrophilic parts. Studies on NIS in AE-induced ALI are limited. We aimed to study the protective effects and mechanisms of NIS in AE-induced ALI.

**Materials and methods:** Five different groups (n = 6 in each group) were created: sham, AE, AE + NIS pretreatment (0.5 mg/kg), AE + NIS pretreatment (1 mg/kg), and AE + post-AE NIS (1 mg/kg). AE-induced ALI was introduced by the infusion of air via the pulmonary artery. Aerosolized NIS were administered via tracheostomy.

**Results:** Pulmonary AE-induced ALI showed destruction of the alveolar cell integrity with increased pulmonary microvascular permeability, pulmonary vascular resistance, pulmonary edema, and lung inflammation. The activation of nuclear factor-κB (NF-κB) increased the expression of pro-inflammatory cytokines, and sodium-potassium-chloride co-transporter isoform 1 (NKCC1). The pretreatment with NIS (1 mg/kg) prominently maintained the integrity of the epithelial lining and suppressed the expression of NF-κB, pro-inflammatory cytokines, and NKCC1, subsequently reducing AE-induced ALI.

**Conclusions:** NIS maintained the integrity of the epithelial lining and suppressed the expression of NF-κB, pro-inflammatory cytokines, and NKCC1, thereby reducing hyperpermeability, pulmonary edema, and inflammation in ALI.

## Introduction

Some invasive iatrogenic procedures, such as radical neck dissection, venous access, angiography, and laparoscopic surgery, may cause air embolism (AE) of the lungs. In addition to these iatrogenic procedures, divers and high-altitude pilots who are exposed to high pressure sometimes experience pulmonary AE. When the amount of air emboli exceeds the absorption capacity of the lungs, it will cause acute lung injury (ALI), which is called pulmonary AE-induced ALI 1. AE-induced ALI often manifests as pulmonary edema, pulmonary hypertension, and severe hypoxia 1. Although AE-induced ALI rarely occurs clinically, once it occurs, it is very likely to lead to death 1, 2. Therefore, it is important to investigate effective therapies for AE-induced ALI.

There are several mechanisms underlying AE-induced ALI 3-5. Pulmonary AE physically obstructs and injures the microvasculature, which results in hyper-permeability of the pulmonary capillaries 3. This process further causes the release of mediators and inflammatory responses 3. Neutrophils interact with AE and release inflammatory mediators, pro-inflammatory cytokines, and proteases that further damage the lungs 4, 5. These factors finally lead to severe damage of the lung tissues, pulmonary edema, and an impaired gas exchange 3-5.

Surfactants are important for lung physiology because of their surface-active properties that help to decrease the alveolar surface tension 6. Previous studies have shown that surface tension with shear force can induce epithelial cell damage 7 which may destroy the alveolar epithelial cell (AEC) integrity. A dysfunction of surfactants results in the loss of aerated volume and a poor ventilation/perfusion match 6. An exogenous surfactant therapy is reported to be effective for ALI 8, 9. However, the cost of producing surfactants from animal lungs or synthetic surfactant analogs is very high, and these are not easy to obtain clinically 10.

Nonionic surfactants (NIS), a kind of surfactants composed of two parts, hydrophilic and hydrophobic parts, do not have electric charge on their hydrophilic parts 11. NIS orient themselves in the layer lattice, where the hydrophobic parts are arranged towards the aqueous bulk, and the hydrophobic parts are arranged in a way that minimizes the interaction with the aqueous bulk. The features of biphasic coupling can minimize the surface tension 11. In addition, NIS, such as tyloxapol, have been shown to have anti-inflammatory effects with a decreasing level of cytokines 12. NIS are inexpensive and used clinically as a vesicle to deliver drugs, such as amphotericin B and glucocorticoids, to the lungs 13, 14. Because of their ability to minimize the surface tension and anti-inflammatory effects, they might be considered as an alternative therapy to surfactants in ALI.

The regulation of alveolar water is important in the formation of pulmonary edema in ALI 15. The sodium-potassium-chloride cotransporter isoform 1 (NKCC1) in the lungs is important in the regulation of the alveolar fluid 15. NKCC1 is located on the basolateral side of the lung epithelium and controls the flow of ions and water into the alveolar cells. One previous study reported that NKCC1 not only regulates lung water, but also lung inflammation 16. Some studies also reported that mice lacking NKCC1 were protected from bacterial pneumonia 16, 17. We previously suggested that a higher NKCC1 level significantly results in a greater severity of ischemia-reperfusion or ventilator-induced ALI 18, 19. However, studies on NKCC1 in AE-induced ALI are still limited.

The alveolar epithelium constitutes an interface with the outside environment and has the function of maintaining a continuous surface for the gas exchange to occur 20. The destruction of AECs leads to changes in epithelial permeability and causes pulmonary edema 20. ALI is characterized by type I alveolar cell necrosis or apoptosis, basement membrane destruction, and protein-rich fluid retention in the alveolar space or interstitium 20. The loss of AEC integrity further results in transepithelial migration of neutrophils and further leads to increases in pro-inflammatory cytokines or mediators in the lungs 21. Although epithelial injury is one of the important mechanisms of ALI 21, studies on the effects of NIS in lung epithelial injuries are still limited. Therefore, we conducted this study to determine the effects of NIS on AEC integrity in AE-induced ALI.

The maintenance of AEC integrity is important for the management of ALI. Maintaining AEC integrity can result in decreased pulmonary permeability, edema, and inflammation in ALI. Surface tension is important for AEC integrity. NIS act by decreasing the surface tension and have anti-inflammatory effects. Therefore, we hypothesized that NIS might decrease pulmonary AE-induced ALI by maintaining lung epithelial integrity and decreasing the level of pro-inflammatory cytokines.

## Materials and methods

### Animal care and procedures of the lung model

The Animal Review Committee of the National Defense Medical Center approved this study. Male Sprague-Dawley (S-D) rats aged 10-12 weeks and weighing 300 - 350 g from BioLASCO Taiwan Co., Ltd were used. These rats were housed at a temperature of approximately 22°C and a humidity of approximately 60% and maintained on a 12-hour light-dark cycle at the Laboratory Animal Center of National Defense Medical Center.

The rats were anesthetized using an intraperitoneal (IP) injection of pentobarbital (50 mg/kg). A tracheostomy was conducted after deep anesthesia was achieved and the rats were connected to a mechanical ventilator (MV). The MV was set at a tidal volume of 3 mL, a breathing frequency of 60 cycles/min, and a positive end-expiratory pressure (PEEP) of 1 cm H_2_O.

The procedures of the isolated lung model were performed after sternotomy and injection of heparin into the right ventricle (RV). We then inserted a cannula in the left atrium (LA) for recirculation. We inserted another catheter into the main pulmonary artery (PA) through the RV. Pulmonary circulation was maintained using a peristaltic pump (Model 1203, Harvard Apparatus). The recirculated perfusate was composed of 10-12 mL of blood mixed (1:1) with a physiological solution and bovine albumin (4 g/dL). The rate of perfusion was maintained at 8 mL/min, and the temperature was maintained at 37°C.

### Experiment protocols

**Figure 1:**
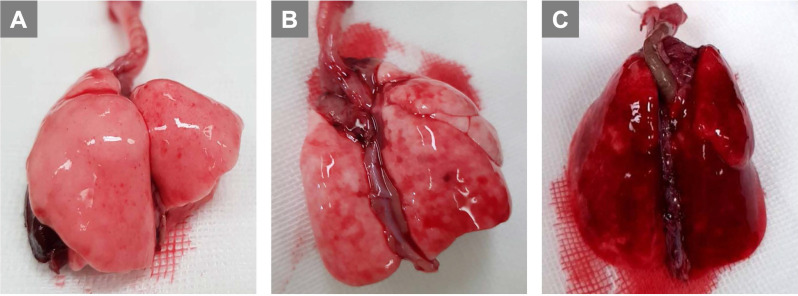
** Titration of air amount via pulmonary artery to induce lung injury.** (A) 0.5 mL (B) 0.7 mL (C) 1.0 mL.

Preliminary experiments with infusions of 0.5 mL, 0.7 mL, and 1.0 mL of air via PA were performed. Grossly, lung injury was not prominent with an infusion of 0.5 mL of air (Fig. 1A). Lung injury was prominent with an infusion of 0.7 mL of air (Fig. 1B), and we were able to complete the further experiments. When infusing 1.0 mL of air (Fig. 1C), we observed that lung injury was too severe with prominent hemorrhage, and we could not perform further experiments. Therefore, for further study, ALI was induced by infusing 0.7 mL of air via PA.

**Figure 2:**
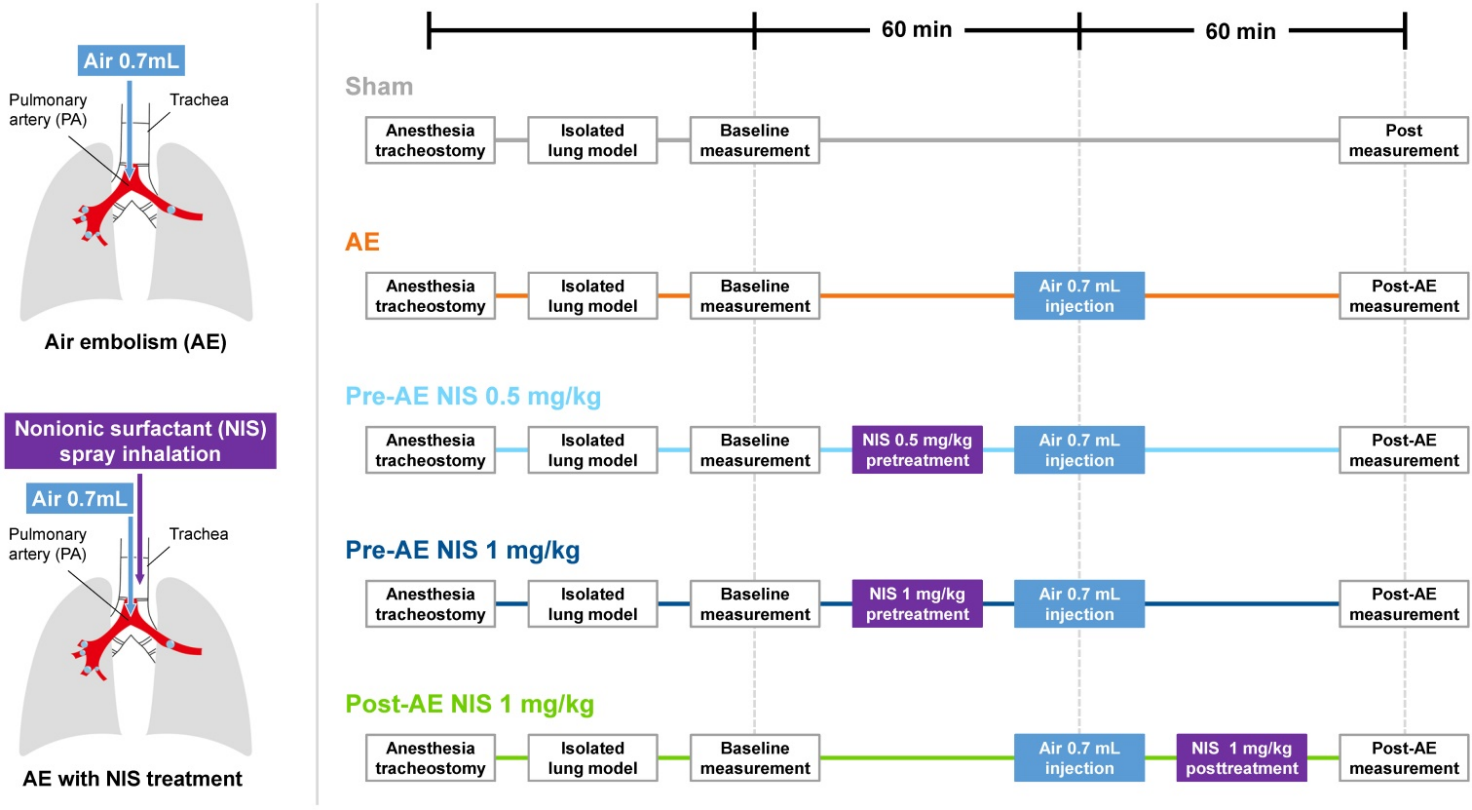
The experimental protocols of air-emboli induced lung injury and NIS treatment.

The experimental protocols are shown in Fig. 2. The rats were divided into five groups (n = 6 per group): sham group, AE group, AE with NIS pretreatment (0.5 mg/kg, Pre-AE NIS 0.5 group), AE with NIS pretreatment (1 mg/kg, Pre-AE NIS 1 group), and AE + post-AE NIS (1 mg/kg, Post-AE NIS 1 group). The rats were randomly assigned to these five groups. The timing of the measurements and the induction of AE are displayed.

NIS (nonyl β-D-glucopyranoside, CAS 69984-73-2 from Santa Cruz Biotechnology) was dissolved well in 70 µL of distilled water, which was then administered into an intratracheal MicroSprayer aerosolizer (IA-1C; Penn-Century, Philadelphia, PA, USA). The sham group received an identical volume of intratracheal distilled-water spray. Baseline measurements were performed after stabilization of the animals. Pre-treatment with NIS before inducing AE of air was then administered to Pre-AE NIS 0.5 group and Pre-AE NIS 1 group. Post-AE measurement was performed 60 minutes after the induction of AE. In Post-AE NIS 1 group, NIS was administered after inducing AE, and post-AE measurement was also performed 60 minutes after the induction of AE (Fig. 2).

### Microvascular permeability

The index of microvascular permeability (Kf) was determined from the changes in lung weight (LW) induced by an elevated pulmonary venous pressure (PVP) 18. During the measurement, the PVP increased rapidly by 10 cm H_2_O for 7 min. The steady phase of LW changes as a function of time (ΔW/ΔT) was plotted on a semi-logarithmic scale. It was then extrapolated to time zero to obtain the initial rate of the trans-capillary filtration. Kf was defined as the y-intercept divided by PVP and LW 18. Kf1 (baseline) and Kf2 (post-AE) were measured. The changes in Kf (%) were calculated as (Kf2 - Kf1)/Kf1.

### Pulmonary arterial pressure (PAP) and pulmonary vascular resistance (PVR)

PAP was monitored during the experiment. The PAP at baseline was recorded as PAP1, and the PAP after AE-induced ALI was recorded as PAP2. The PVR was calculated as the difference in pressures between PAP and PVP, divided by the perfusate flow rate (Q): PVR= (PAP - PVP)/Q. The PVR1 (baseline) and PVR2 (post-AE) were calculated.

### Pulmonary edema

At the end of the experiment, we removed and weighed one part of the right middle lobe (RML) and then dried it at 60 °C for 48 h. We calculated the lung wet/dry (W/D) ratio by dividing the wet and dry weights of the lungs.

### Alveolar fluid clearance (AFC) measurement

The lungs were inflated, and followed by administration of 12.5 mL/kg body weight (BW) instillate containing fluorescein isothiocyanate (FITC)-conjugated albumin (Sigma-Aldrich, St. Louis, MO, USA) for one minute 5. We aspirated the alveolar fluid (100 μL) at 1 and 15 mins after instillation. We centrifuged the aspirate at 3,000 × *g* for 10 mins. We then measured the fluorescence activity in the supernatant. AFC was calculated from the changes in albumin concentration of the alveolar fluid: AFC = (Cf - Ci)/Cf × 100, where Ci represents the initial concentration of FITC-albumin and Cf represents the final concentration of FITC-albumin.

### Histopathology of the lung tissues

After the experiment, the lungs were infused with 10% formaldehyde through the trachea at 20 cm H_2_O. We immersed the lung tissues in formaldehyde for 24 h and then embedded the tissues in paraffin wax. The lung tissues were then cut into 4 - 6-µm-thick sections, followed by staining of the tissues with hematoxylin and eosin (H&E).

### Tissue neutrophil quantification

We used the H&E-stained sections to count the neutrophils 22. We have identified neutrophils based on the following characteristics: neutrophils have very characteristic nuclei with 3-5 lobes connected by thin strands; neutrophils have a rich pink cytoplasm with coarser granules in the cytoplasm and the size of neutrophils is about 10-15 µm, between lymphocytes and monocytes. The pathologist proved the identification of neutrophils. Neutrophils were counted on each slide in ten random high-power fields (HPF, 400 ×) 18.

### Immunofluorescent staining of alveolar epithelial type I cells

We further performed immunofluorescent staining (Novus Biologicals, USA) for alveolar type I cells. The sections were treated with 3% hydrogen peroxide, and nonspecific binding sites were blocked with bovine serum albumin (BIONOVAS, Toronto, Canada) for 30 mins. The sections were incubated with antibodies against rat type I cell 40-kDa protein (RTI40)/type I cell alpha (T1α) protein (dilution 1:50; Sino Biological, USA) and NKX2.1/type II (NKX2.2) (dilution 1:100; NOVUS Biological, Toronto, Canada) overnight at 4°C. The fluorescent label-conjugated FITC- and Texas Red-labeled secondary antibody (Jackson Immunoresearch Laboratories) was diluted with 1% bovine serum albumin in phosphate buffered saline at a ratio of 1:250 at room temperature for 1 h, respectively. We evaluated the sections using an EVOS M7000 Imaging System (Thermo Fisher Scientific, USA).

### Quantification of AEC discontinuation

We used immunofluorescent staining sections of T1α-positive cells to perform quantified and statistical analysis. We counted AEC discontinuations in each group of ten random high-power fields (HPF, 400 ×). The mean count of sham group is used as the denominator to calculate the fold of counts of other groups.

### Levels of pro-inflammatory cytokines in the perfusate and lung tissues

The expression of pro-inflammatory cytokines in the perfusate and tissues, including interleukin-1ß (IL-1β), tumor necrosis factor-α (TNF-α), and C-X-C motif ligand 1(CXCL-1), was determined using a commercial Enzyme-linked immunosorbent assay (ELISA, R&D Systems Inc., Minneapolis, MN).

### Immunoblotting of phosphorylated nuclear factor-κB p65, IκB-α, and NKCC1

Nuclear and cytoplasmic proteins were extracted from the lungs using a nuclear/cytosol extraction kit (BioVision, Inc., Mountain View, CA). The blots were incubated with antibodies against phosphorylated nuclear factor-κB (NF-κB) p65, inhibitor of NF-κB alpha (IκB-α), and NKCC1 (Cell Signaling Technology, Danvers, MA) overnight at 4 °C. Enhanced chemiluminescence reagents were used to visualize the bands, that were then exposed to radiography films. The blots were stripped using an anti-TATA antibody or anti-β-actin antibody to ensure equal loading.

### Data analysis

We analyzed the data using Prism 9 software (GraphPad Software, LLC., San Diego, CA, USA). Kruskal-Wallis test and Tukey's multiple comparison test were performed for *post-hoc* comparisons and intergroup differences. A statistically significant difference was set at a *p* < 0.05.

## Results

### NIS decreased air emboli-induced microvascular hyperpermeability, pulmonary hypertension, pulmonary vascular resistance, lung water, and maintained AFC

**Figure 3:**
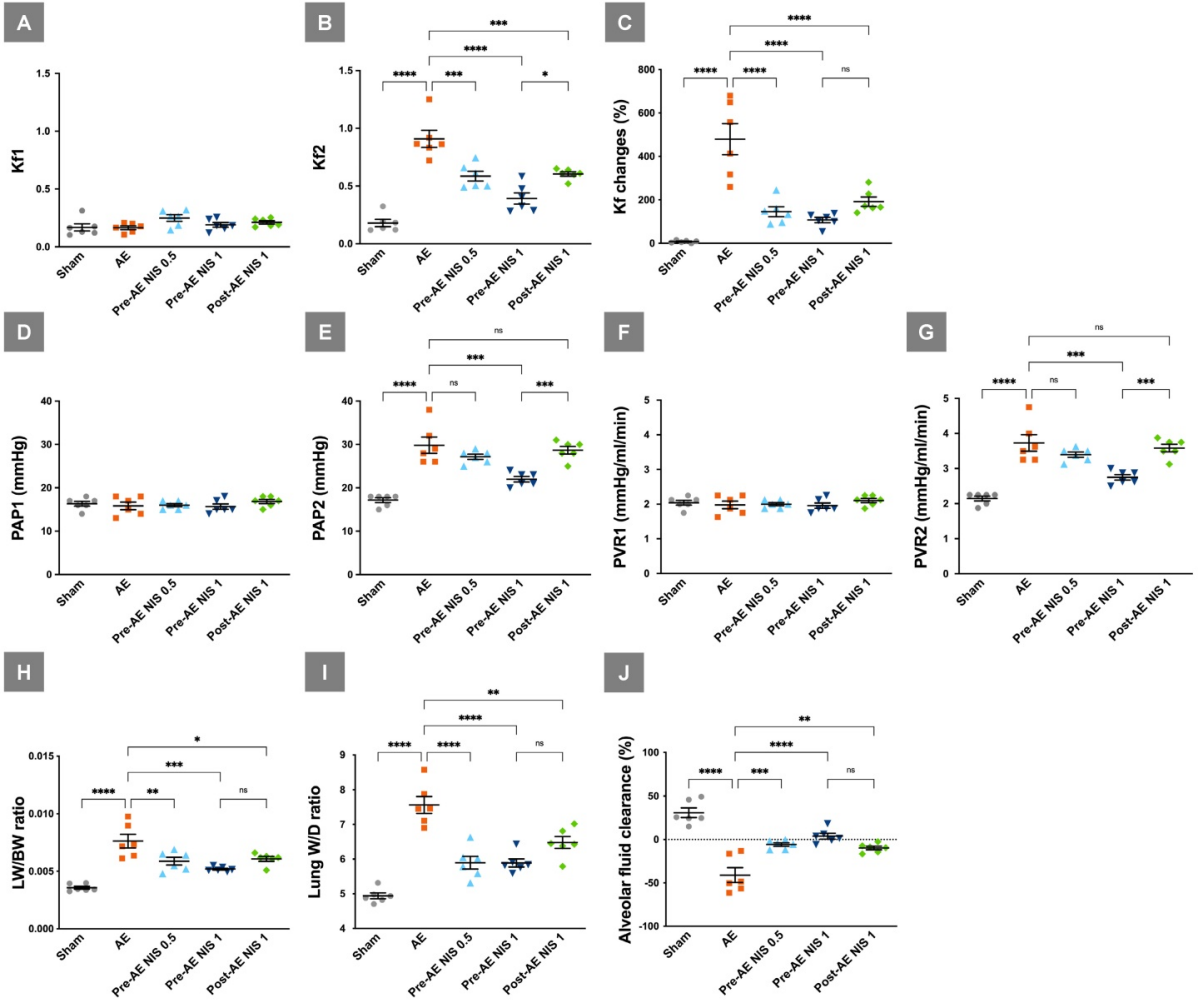
** NIS decreased air emboli-induced microvascular hyperpermeability, pulmonary hypertension, pulmonary vascular resistance, lung water, and maintained AFC.** Kf1 (A) was not significantly different among the groups, and Kf2 (B) was increased in the AE group. NIS administration before and after AE decreased post-AE microvascular permeability. (C) Changes in Kf were prominent in the AE group and were decreased in rats pretreated with 0.5 and 1 mg/kg NIS, and posttreated with 1 mg/kg NIS. (D) The baseline PAP1 was similar among the five groups, while post-AE PAP2 (E) was increased in the AE group compared with the sham group. NIS (1 mg/kg) administration before AE decreased PAP2, compared to the other AE groups. (F) The baseline PVR1 was similar among the five groups, while post-AE PVR2 (G) was increased in the AE group compared with the sham group. NIS (1 mg/kg) administration before AE decreased PVR2 compared to the AE groups. (H) LW/BW and (I) W/D were increased in the AE group. Pretreated with 0.5 and 1 mg/kg NIS pretreatment, and 1 mg/kg NIS posttreatment decreased LW/BW and W/D. (J) AFC was decreased in the AE group. Pretreated with 0.5 and 1 mg/kg NIS pretreatment and 1 mg/kg NIS posttreatment increased AFC. ns: *p* > 0.05, not significant; *p < 0.05; **p < 0.01; ***p < 0.001; ****p < 0.0001.

The baseline Kf1 was similar among the five groups (*p* > 0.05, Fig. 3A), while the post-AE Kf2 levels (Fig. 3B) were increased in the AE group, compared with the sham group (*p* < 0.05). NIS administration before (0.5 and 1 mg/kg) and after (1 mg/kg) AE decreased the Kf2 level compared to the AE group (*p* < 0.05). Changes in Kf (Fig. 3C) were prominent in the AE group, compared with the sham group (*p* < 0.05) and were attenuated in the rats pretreated with 0.5 and 1 mg/kg NIS (*p* < 0.05).

The baseline PAP1 was similar among the five groups (*p* > 0.05, Fig. 3D), while post-AE PAP2 was increased in the AE group compared with the sham group (*p* < 0.05). NIS (1 mg/kg) administration before AE decreased PAP2, compared to the other AE groups (*p* < 0.05, Fig.3E). The baseline PVR1 was similar among the five groups (*p* > 0.05, Fig. 3F), while post-AE PVR2 was increased in the AE group compared with the sham group (*p* < 0.05). NIS (1 mg/kg) administration before AE decreased PVR2 compared to the other AE groups (*p* < 0.05, Fig. 3G).

LW/BW (Fig. 3H) and the lung W/D ratio (Fig. 3I) were increased in the AE group compared with the sham group (*p* < 0.05). Pretreatment with 0.5 and 1 mg/kg NIS and posttreatment with 1 mg/kg NIS significantly decreased the pulmonary edema, compared with the AE group (*p* < 0.05). AFC was markedly decreased in the AE group, compared with the sham group (*p* < 0.05). Pretreatment with 0.5 and 1 mg/kg NIS and posttreatment with 1 mg/kg NIS significantly increased AFC, compared with the AE group (*p* < 0.05, Fig. 3J).

### NIS attenuated lung injury and neutrophilic sequestration

**Figure 4:**
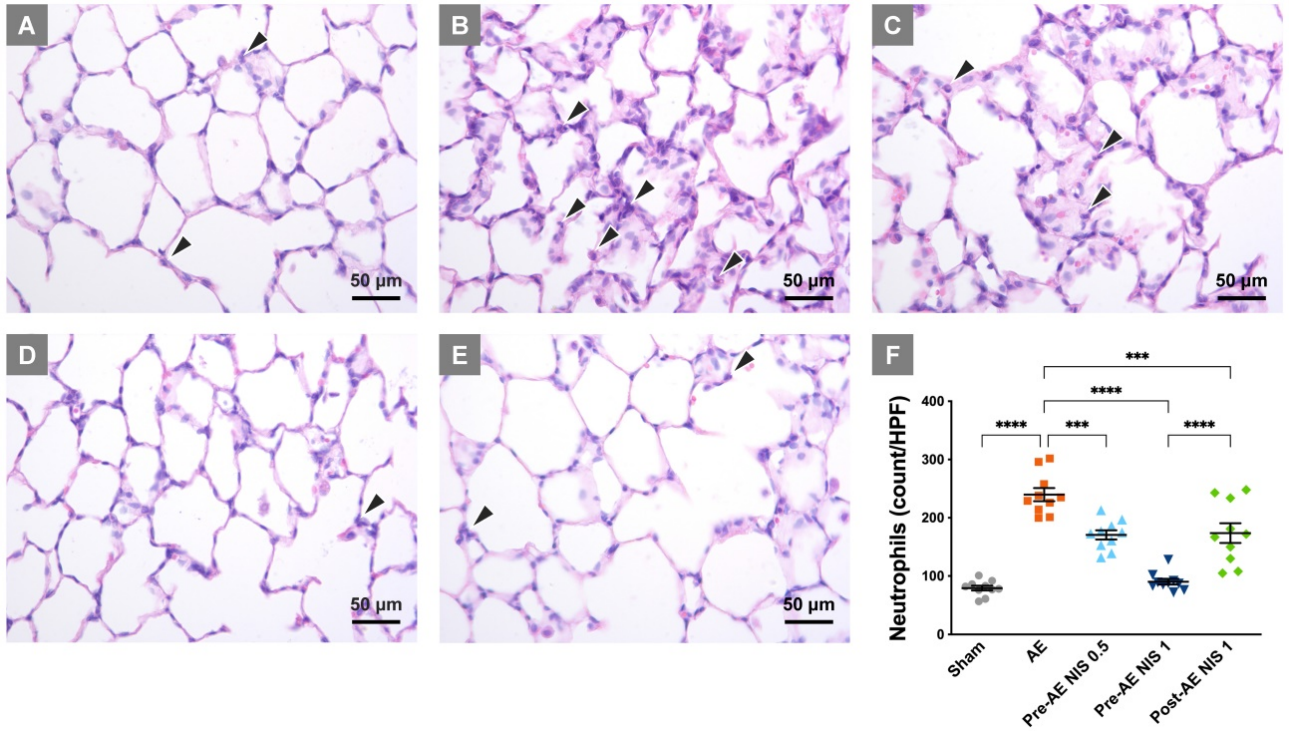
** NIS decreased lung injury and neutrophilic sequestration.** The rats in the sham group (A) presented with normal histology, while those in the AE group (B) showed neutrophilic sequestration and interalveolar septum thickening. The severity of lung injury was significantly reduced in rats that were pretreated with 0.5 mg/kg NIS (C) and were markedly attenuated by 1 mg/kg NIS pretreatment (D). Post-AE NIS (1 mg/kg) also decreased the severity of lung injury (E). There was a notable increase in neutrophils in the AE group (F), and the neutrophilic counts were significantly reduced by pretreatment and post-AE NIS. The best effect was NIS pretreatment at 1.0 mg/kg. The neutrophils are labeled with black arrowheads. HPF: high-power fields. ***p < 0.001; ****p < 0.0001.

The rats of the sham group presented with relatively normal histology (Fig. 4A), while the sections in the AE group showed a prominent inter-alveolar septum thickening and neutrophilic infiltration in lung tissues (Fig. 4B). The severity of lung injury was reduced in rats that were pretreated with 0.5 mg/kg NIS (Fig. 4C), 1 mg/kg (Fig. 4D), and post-AE NIS (1 mg/kg, Fig. 4E).

The quantified neutrophilic count (Fig. 4F) demonstrated a notable increase in the neutrophilic counts in the AE group, compared with the sham group (*p* < 0.05) and was significantly reduced by 0.5 and 1 mg/kg NIS pretreatment and 1 mg/kg post-AE NIS, compared with the AE group (*p* < 0.05). The effect of 1 mg/kg NIS pretreatment was better than the pretreatment with 0.5 mg/kg or 1 mg/kg post-AE surfactant (*p* < 0.05).

### NIS attenuated injury of alveolar epithelial type I cells

**Figure 5:**
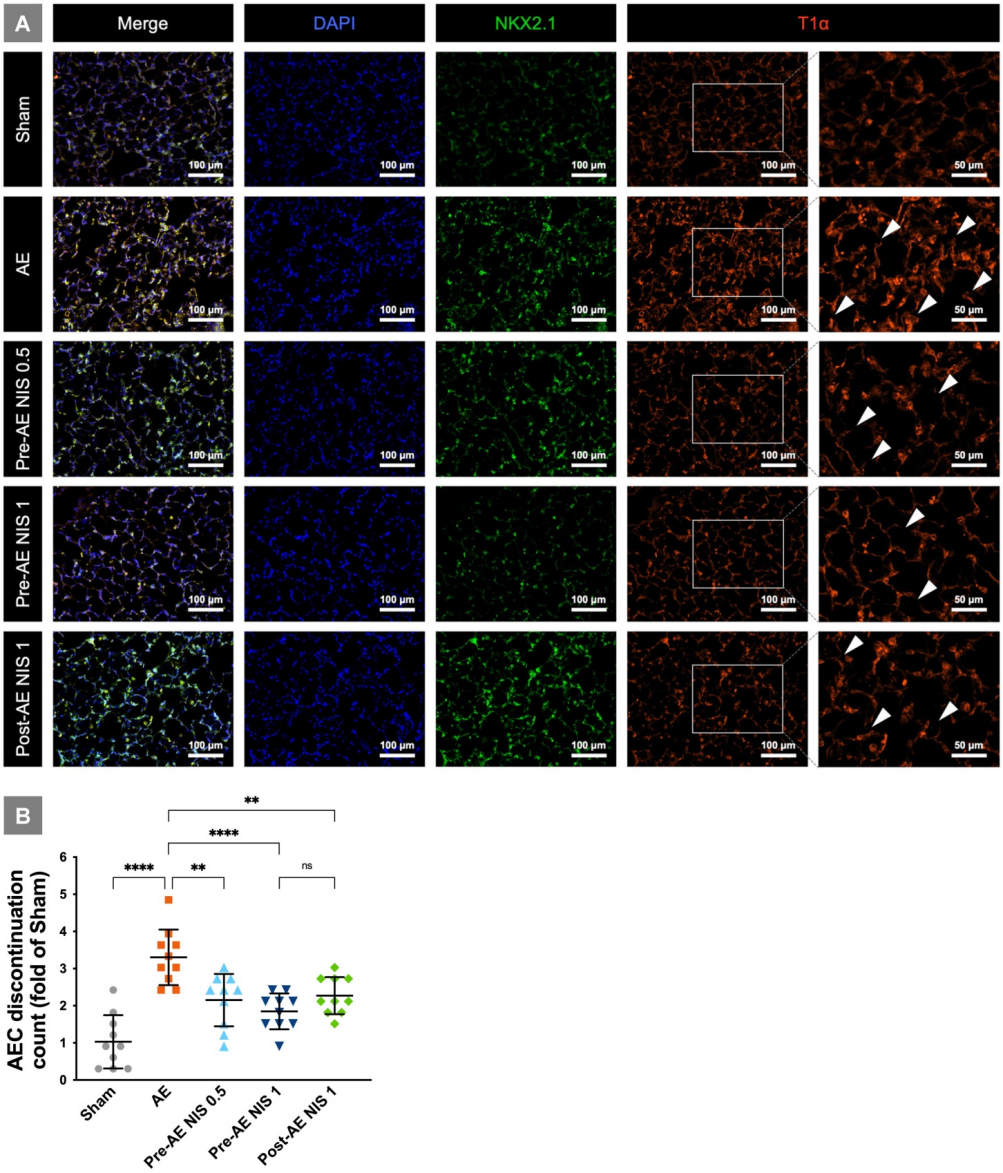
** NIS attenuated alveolar epithelial cell injury.** (A) The rats in the sham group had intact alveolar epithelial cells and histoarchitecture. The rats in the AE group had a destroyed AEC continuation (white arrowheads). AEC continuation was partially maintained in the rats pretreated and post-treated NIS. The integrity was better maintained in rats pretreated with 1 mg/kg NIS than pretreated 0.5 mg/kg NIS and post-treated 1 mg/kg NIS. T1α-positive cells (red) represented alveolar epithelial type I cell. NKX2.1-positive cells (green) represented alveolar epithelial type II cell. DAPI-positive (blue) cells represented nucleus. (B) AEC discontinuation count (folds of count to sham group) based on T1α-positive cells. There was a notable increase in discontinuation in the AE group, and the discontinuation counts were significantly reduced by pre-AE and post-AE NIS. The effect was better in pre-AE 1 mg/kg NIS than pre-AE 0.5 mg/kg NIS and post-AE 1 mg/kg NIS. **p < 0.01; ****p < 0.0001.

The integrity of alveolar epithelial type I cells is presented in Fig. 5A. The rats in the sham group showed intact alveolar epithelial cells and histoarchitecture. The rats in the AE group had a destroyed AEC continuation (white arrowheads), which indicated alveolar epithelial cell damage. AEC continuation was partial maintained in the rats that were pretreated with 0.5 mg/kg NIS and post-treated with 1 mg/kg NIS. The integrity was better maintained in rats pretreated with 1 mg/kg NIS. The AEC discontinuation counts (fold of counts to sham group) based on T1α-positive cells is presented in Fig. 5B. There was a notable increase in discontinuation in the AE group, and the discontinuation counts were significantly reduced by pre-AE and post-AE NIS (all p<0.05). The effect was better in pre-AE NIS 0.5mg than that in post-AE NIS 1 mg (p<0.05).

### NIS decreased the expression of IL-1β, TNF-α, and chemokine CXCL-1

**Figure 6:**
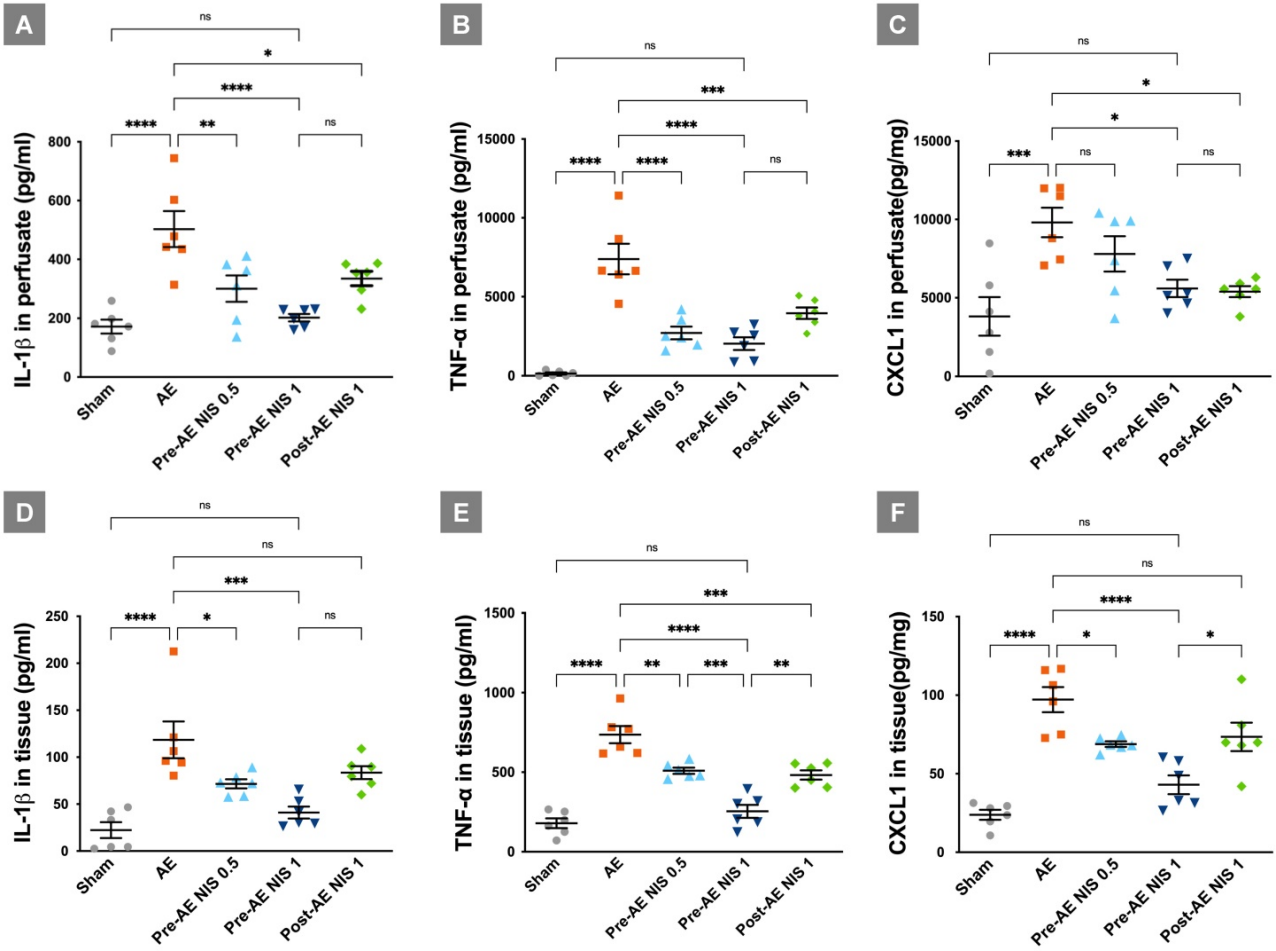
** NIS decreased the expression of proinflammatory cytokines.** IL-1β in perfusate (A) was reduced only after 1 mg/kg NIS pretreatment (*p* < 0.05). TNF-α in perfusate (B) was reduced after 0.5 mg/kg and 1 mg/kg NIS pretreatment (*p* < 0.05). CXCL-1 in perfusate (C) was reduced after 1.0 mg/kg NIS pretreatment and post-AE 1.0 mg/kg NIS (*p* < 0.05) treatment. IL-1β in lung tissues (D) was reduced only after 1 mg/kg NIS pretreatment (*p* < 0.05). TNF-α in lung tissues (E) was reduced after 0.5 mg/kg, 1 mg/kg NIS pretreatment, and post-AE NIS at 1.0 mg/kg (*p* < 0.05). CXCL-1 in lung tissues (F) was reduced only after 1 mg/kg NIS pretreatment (*p* < 0.05). ns: *p* > 0.05, not significant; *p < 0.05; **p < 0.01; ***p < 0.001; ****p < 0.0001.

The expressions of cytokines and chemokine are presented in Figure 6. The levels of IL-1β (Fig. 6A), TNF-α (Fig. 6B), and CXCL-1 (Fig. 6C) in the perfusate were increased in the AE group, compared with the sham group (*p* < 0.05). IL-1β in the perfusate was reduced after administration of 0.5 mg/kg and 1 mg/kg NIS pretreatment, and 1 mg/kg NIS posttreatment, compared with the AE group (*p* < 0.05). TNF-α in the perfusate was reduced after administration of 0.5 mg/kg and 1 mg/kg NIS pretreatment, and 1 mg/kg NIS posttreatment, compared with the AE group (*p* < 0.05). CXCL-1 in the perfusate was reduced after administration of 1 mg/kg NIS pretreatment, and 1 mg/kg NIS posttreatment, compared with the AE group (*p* < 0.05).

The expression levels of IL-1β (Fig. 6D), TNF-α (Fig. 6E), and CXCL-1 (Fig. 6F) in the lung tissues showed a similar pattern to that in the perfusate. These pro-inflammatory cytokines in lung tissues were increased significantly in the AE group, compared with the sham group (*p* < 0.05). IL-1β in the lung tissues was reduced after administration of 0.5 mg/kg and 1 mg/kg NIS pretreatment, compared with the AE group (*p* < 0.05). TNF-α in the lung tissues was reduced after administration of 0.5 mg/kg and 1 mg/kg NIS pretreatment, and 1 mg/kg NIS posttreatment, compared with the AE group (*p* < 0.05). Moreover, the effect of 1 mg/kg NIS pretreatment was better than the 0.5 mg/kg NIS pretreatment and 1 mg/kg post-AE NIS, compared with 1 mg/kg NIS pretreatment (*p* < 0.05). CXCL-1 in the lung tissues was reduced after administration of 0.5 mg/kg and 1 mg/kg NIS pretreatment, compared with the AE group (*p* < 0.05).

### NIS decreased the air emboli-induced expression of nuclear phosphorylated NF-κB and NKCC1 pathways

**Figure 7:**
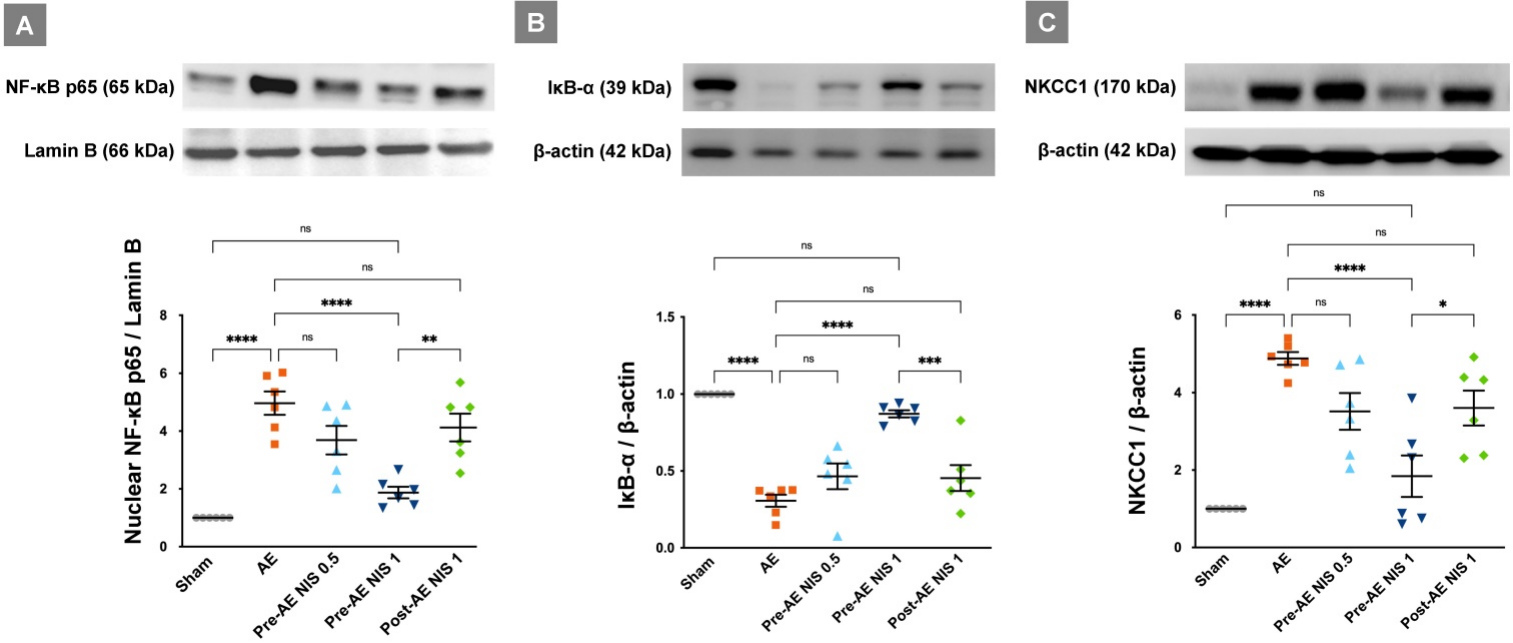
** NIS decreased air emboli-induced NF-κB activation and NKCC1 expression.** There was a higher level of nuclear phosphorylated NF-κB p65 (A) in the AE group, along with a significant suppression of IκB-α (B) levels (*p* < 0.05). Only 1 mg/kg NIS pretreatment reduced NF-κB p65 levels and increased IκB-α (*p* < 0.05). NKCC1 (C) levels were increased in the AE group (*p* < 0.05) and only the pretreatment with 1 mg/kg NIS decreased NKCC1 levels (*p* < 0.05). ns: *p* > 0.05, not significant; *p < 0.05; **p < 0.01; ***p < 0.001; ****p < 0.0001.

In the AE group, the levels of nuclear phosphorylated NF-κB p65 were increased (Fig. 7A), and the levels of IκB-α (Fig. 7B) were decreased, compared with those in the sham group (*p* < 0.05). Only 1 mg/kg NIS pretreatment reduced the nuclear phosphorylated NF-κB p65 levels and restored those of IκB-α (*p* < 0.05). NIS pretreatment at 0.5 mg/kg and post-AE surfactant at 1 mg/kg reduced the phosphorylated NF-κB p65 levels and restored IκB-α levels but was not statistically significant compared with those of the AE group (*p* > 0.05).

The levels of NKCC1 were significantly increased in the AE group (*p* < 0.05, Fig. 7C). Pretreatment with 1 mg/kg NIS decreased the levels of NKCC1 (*p* < 0.05). There were no differences in NKCC1 expression between the pretreatment with NIS 0.5 mg/kg and post-AE 1 mg/kg, compared with the AE group (*p* > 0.05).

## Discussion

AE-induced lung injury showed severe lung inflammation, high PVR, pulmonary edema, microvascular hyper-permeability, and alveolar type I cell injury. AE-induced ALI also presented with an increased expression of pro-inflammatory cytokines, NF-κB, and NKCC1. NIS attenuated AE-induced AEC injury by maintaining its integrity and decreasing pulmonary edema, PVR, hyper-permeability, lung inflammation, expression of pro-inflammatory cytokines, NF-κB, and NKCC1.

AEC integrity is important in the pathogenesis of lung injury. The AEC barrier prevents the exudation of protein-rich fluid from capillaries into the alveolar spaces 23. Pulmonary vascular permeability is primarily determined by epithelial and endothelial barriers 24. Damage to the AEC integrity results in increasing permeability and influx of protein-rich fluid into the alveolar space 23. This further results in the recruitment of neutrophils into the alveolar space 23. Finally, damage that compromises AEC integrity leads to severe pulmonary edema and inflammation. Therefore, AEC integrity and pulmonary hyperpermeability are important therapeutic issues in ALI. In the current study, we revealed the destruction of AEC integrity with concurrent pulmonary hyper-permeability in AE-induced ALI. The administration of NIS could maintain AEC integrity, decreased pulmonary hyper-permeability, and decreased neutrophil sequestrations.

Surface tension with shear force can induce epithelial cell damage and destroy AEC integrity 7. During ALI, water molecules in the alveoli cause greater surface tension 25, which causes further alveolar injury and the loss of AEC integrity 7. These results further aggravate lung injury. The phospholipid structures of the hydrophobic parts of NIS stabilize the phospholipid structure and make it face the air space 11. This further results in a reduced surface tension, maintaining the air-water interface and, therefore, preventing the alveoli from further injury. Therefore, by decreasing the alveolar surface tension, NIS may maintain AEC integrity.

Neutrophilic influx into the lungs is an important pathogenesis mechanism of ALI 26. Neutrophils are present in three pools within the body: bone marrow pool, venous marginal pool, and the pool within the tissues 27. This was a study of an isolated lung model. Therefore, neutrophils did not originate from the venous marginal pool or the bone marrow. However, there was still neutrophil pooling within the lung tissues. In addition, the re-circulated perfusate was composed of blood mixed (1:1) with a physiological salt solution. Therefore, the neutrophils participating in ALI are derived from the pool within the lungs and the blood in the perfusate.

Here, we confirmed that NIS decreased cytokines and neutrophil sequestration in AE-induced ALI. NF-κB regulates the genes of pro-inflammatory cytokine and is, therefore, important in ALI 28. NF-κB activation typically results in an increased expression of pro-inflammatory cytokines, which have a chemotactic effect and induce the migration of neutrophils into the alveoli. Migratory neutrophils further degranulate and release cytotoxic enzymes that damage the lung tissues 29. Our results showed that NF-κB activation, the expression of pro-inflammatory cytokines, and neutrophil sequestration in AE-induced ALI were decreased by NIS treatment. In a previous study on NIS, Pluronic F68 was also reported to inhibit the adherence and migration of neutrophils 30.

Studies on NIS nonyl β-D-glucopyranoside in ALI are lacking. There are some studies on NIS, such as tyloxapol and pluronic F68 12, 30. Tyloxapol was shown to inhibit the expression of IL-1, IL-6, and IL-8 12. In addition, tyloxapol is a potent antioxidant that effectively scavenges the oxidant hypochlorous acid 31 and protects against hyperoxic ALI 31. Pluronic F68 reduced the lavage neutrophil, monocyte, and macrophage counts in rats with bleomycin-induced ALI 30. It is also proposed that Pluronic F68 may be able to interact with the alveolar membrane due to its mixed hydrophilic and lipophilic properties 30. In our current study, we also suggested that nonyl β-D-glucopyranoside has anti-inflammatory effects and can decrease the severity of pulmonary AE-induced lung injury.

The role of NIS in alveolar ion channels and AFC requires further elucidation. In this study, we revealed that NIS decreased NKCC1 levels and maintained the AFC levels after lung injury. NKCC1 is important in the regulation of lung fluids and inflammation 15. NF-κB activation causes osmotic stress and cellular swelling 32. These proteins then activate with-no-lysine kinase and NKCC1 32. A higher expression of NKCC1 results in the dysregulation of the water transport system, leading to cellular swelling, inflammation, and damage 16. Here, we confirmed that AE-induced ALI presented with high NKCC1 and impaired AFC. NIS-associated inhibition of the NF-κB cascade decreases the expression of NKCC1 and, therefore, restores AFC 5. In addition, NIS reduces the alveolar surface tension and prevents lung fluids from leaking into the alveolar space 33. Therefore, the administration of NIS can restore AFC and decrease the severity of lung injury.

### Clinical implications

Pulmonary AE is a fatal complication that usually requires intensive care. It has high mortality and morbidity rates. Therefore, it is important to study the effective treatment of AE-induced ALI. Surfactants have an important physiological function to maintain the surface tension of alveoli. In the current study, we also suggested that NIS can maintain the epithelial integrity, decrease the pulmonary microvascular permeability, edema, and inflammation. Therefore, we suggest the administration of NIS before or after AE as an option to attenuate lung injury. It enables clinicians to address the therapeutic effects of NIS in AE-induced ALI in further clinical research.

Before clinical research, the issue of toxicity should be addressed. Clinically, NIS vesicles have been used for the pulmonary delivery of glucocorticoids, such as beclomethasone dipropionate (BDP), for the treatment of asthma and chronic obstructive pulmonary disease 14. This showed that BDP-loaded NIS had a prominent anti-inflammatory activity 14. Further, NIS did not have a significant cytotoxic activity in human lung cells according to this study 14. One phase I study of intravenous infusion of a NIS, poloxamer 188, in the treatment of patients with acute chest syndrome, also showed no evidence of renal toxicity or other limiting adverse events 34. It is suggested that this NIS is safe to administer to human beings.

### Limitations of the study

There are some limitations to the current study. First, this study was performed in small animals (rats) in an isolated lung model. Although the protective mechanism is rational for the administration of NIS, further studies in large animals or clinical studies in human beings are still necessary. The second limitation is that the experiment was performed in the very early stage of ALI. Further studies on the long-term effects of NIS are necessary. Damage that compromises AEC integrity has also been reported to be associated with lung injury-related fibrosis 23. The effects of NIS on long-term outcomes, such as post-ALI fibrosis, should be confirmed.

## Conclusions

**Figure 8:**
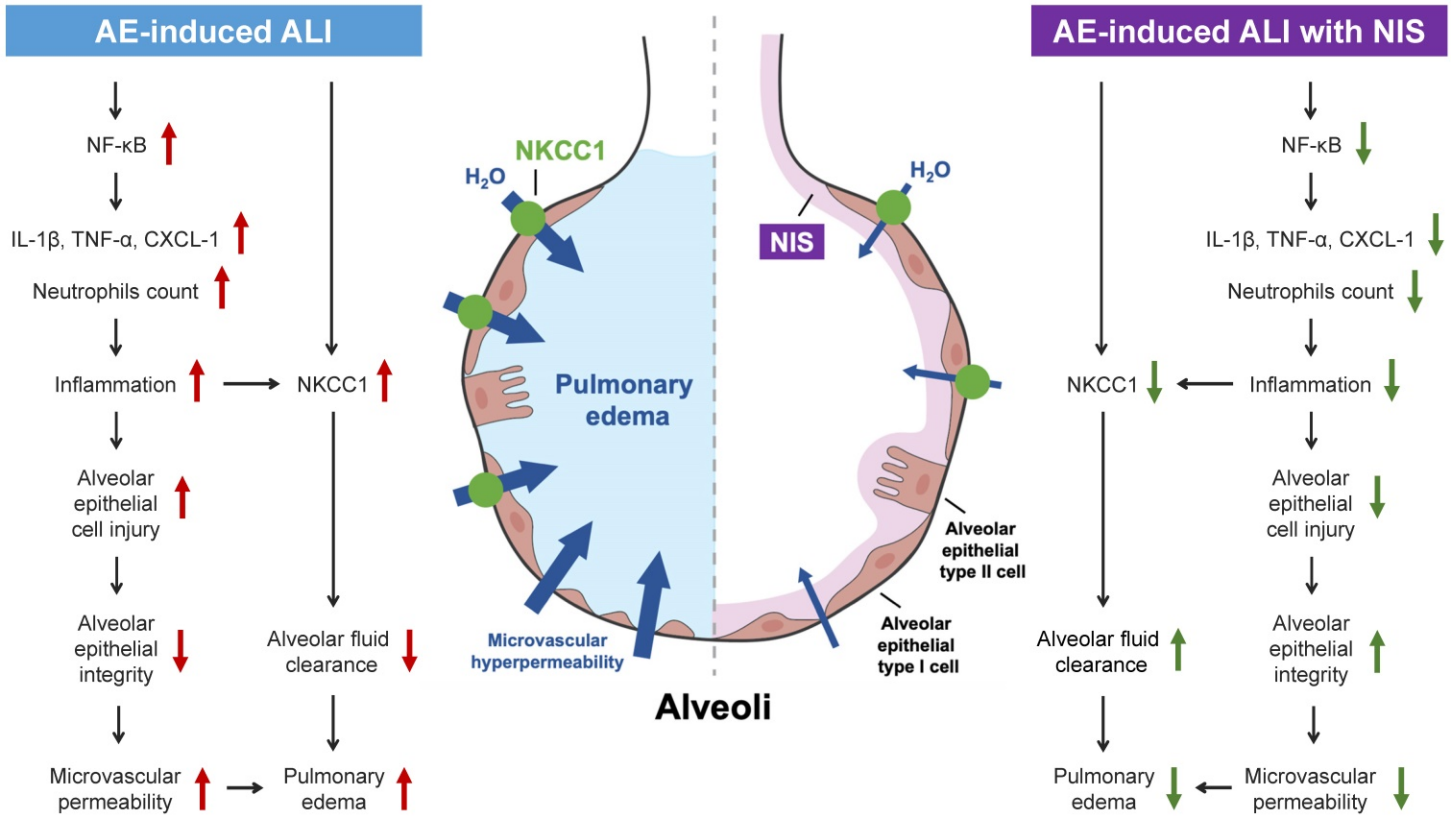
Mechanisms of AE-induced lung injury and protective role of NIS.

AE-induced ALI presents with pulmonary edema, high PVR, alveolar epithelial cell injury, pulmonary microvascular hyperpermeability, lung inflammation, and expression of pro-inflammatory cytokines, the NF-κB pathway, and NKCC1 (Fig. 8, left panel). Pretreatment or post-AE administration of NIS maintained AEC integrity, attenuated lung inflammation and edema, and downregulated pro-inflammatory cytokines, the NF-κB pathway, and NKCC1 expression (Fig. 8, right panel). This study discusses the benefits of NIS in pulmonary AE-induced lung injury. However, further studies are necessary to address the effects of NIS administration in a clinical study.

## Abbreviations

AE: air embolism
ALI: acute lung injury
BW: body weight
CXCL-1: C-X-C motif ligand 1
FITC: fluorescein isothiocyanate
H&E: hematoxylin and eosin
HPF: high-power fields
ICU: intensive care units
IκB-α: inhibitor of NF-κB alpha
IL-1β: interleukin-1ß
IP: intraperitoneal
Kf: microvascular permeability
LA: left atrium
LW: lung weight
RML: right middle lobe
MV: mechanical ventilator
NF-κB: nuclear factor-κB
NIS: nonionic surfactant
NKCC1: sodium-potassium-chloride co-transporter isoform 1
PA: pulmonary artery
PAP: pulmonary arterial pressure
PEEP: positive end-expiratory pressure
PVP: pulmonary venous pressure
PVR: pulmonary vascular resistance
RV: right ventricle
S-D: Sprague-Dawley
TNF-α: tumor necrosis factor-α
W/D: wet/dry
